# The Hospital Nursing Supplement

**Published:** 1893-09-09

**Authors:** 


"The Hospital" Nursing Mirror.

Being the Extra Nursing Supplement of "The Hospital" Newspaper.

[Contributions for this Supplement should be addressed to the Editor, The Hospital, 428, Strand, London, W.C., and should have the word "Nursing" plainly written in left-hand top corner of the envelope.]

### News from the Nursing World.

### WORKHOUSE SICK WARDS AND POOR LAW INFIRMARIES.

It is entertaining to notice the ignorance of certain scribes who presume to write on nursing subjects. We called attention in the *Pall Mall Gazette* to the "abuses and errors connected with the sick wards of some workhouses in this country," and immediately the little coterie of cranks who try to pose as prophets in the world of nursing rise up and "emphatically deny the truth of this wholesale condemnation"—of sick wards? Not at all, but "of the management of our workhouse infirmaries." The veriest tyro in nursing matters, we should have imagined, knew the difference between a Poor Law infirmary and a sick ward in a workhouse. The Poor Law infirmary is often as efficient as it is possible to make any hospital, whilst the sick ward of a workhouse is often at best a relic of barbarism.

### LADIES IN THE NURSERY.

We have received several inquiries lately, respecting the training of ladies as Children's Nurses. There is a very great deal to be said in favour of educated women as nurses from the children's point of view. Early impressions are highly important, and if properly and carefully trained also, a lady should be a much more desirable nurse than a more ignorant woman. From the ladies' point of view, to those who have patience and love for children, the occupation, where a nursemaid is kept, embraces hardly any rough work. The constant duty required, however, should not be overlooked, as it may prove at first very irksome. Still it is not more than is required of the nursery governess, who is often less considered and worse paid than a nurse.

### WHOLESOME WORKERS.

After a short visitation of tropical heat, how gladly most British Islanders welcomed a grey sky and a little wind and rain, in short the familiar cool weather which braces us all up to work, as most of us have to do, whatever the thermometer may register. An interview with a sober-minded, earnest nurse is almost as refreshing as an encounter with a wholesome breeze, especially just now when excitement and affectation are more than usually visible. The faithful, conscientious worker finds "the trivial round, the common task" all sufficing. She clings to the career which she entered upon thoughtfully, and she pursues it with no doubt of its being one of the noblest to which a woman's life can be devoted. To such as these the present suggestion that nursing is a profession to adopt, because it promises freedom and recreation not easily attainable in country homes or strict households, comes as a novel and contemptible suggestion. Nursing, above all things, calls for single-minded devotion, and neither it nor any other work is well done by those whose thoughts are chiefly occupied by plans for the hours or days in which they can escape from the duties of the high calling which they are often unworthy to follow.

### TRUE GENTLE-WOMEN.

"Comparisons are odious," say old-fashioned copybooks, and surely they are right. To distinguish between nurses as "the ordinary" and "the higher educated class" is invidious. They should rank by their merits and their work alone, and surely any other comparisons are obsolete. Amongst Rural as well as Queen's Nurses, a large proportion are, in every sense of the word, gentlewomen, but they do not consider it necessary to assert the fact. The Metropolitan and National Association is now the Central Training School for the Queen's Jubilee Nurses, who are experienced and educated women, and so are the Rural District Nurses, who do equally good work in their special sphere of usefulness. In fact, it is generally conceded that refinement and gentleness constitute the only "first class" in nursing.

### FELLOW-LODGERS.

"This child ought to go to the seaside at once," says many a doctor to many an anxious parent. Perhaps the kind of place most suitable for the case is then mentioned, and, the interview ended, the search for lodgings begins. At home the delicate child has had a bed, and perhaps a room to himself; clean carpets, nice furniture, and well-cooked nourishment, even though the latter may have failed to tempt his capricious appetite. At the seaside "in the season," which is the euphonious term for the time when every place is closely packed with humanity, an invalid must often be content with very inferior accommodation. The crowds of persons who swarm into every well-known watering-place, come from all quarters and from every kind of home, and they are not invariably either clean or healthy. Therefore, in the search for apartments, the parents of a delicate child have many things to consider, and not amongst the least important ranks the question of fellow-lodgers! When half a dozen adults and children are crowded into one bed-room, and several persons are sleeping in the basement in dangerously close proximity to the provisions of the whole establishment, it becomes a matter of vital importance to inquire of the landlady, "Who else have you in the house?"

### TRAINING AT ST. MARYLEBONE INFIRMARY.

To the probationers and nurses at this infirmary a weekly lecture is delivered during six months of the year by Mr. Lunn, the Medical Superintendent, on medical and surgical subjects, the chemistry of food, &c. Clinical instruction is given in the wards, individual instruction in prescriptions, action of drugs, and the general treatment of patients. Classes are held throughout the year for senior and junior probationers respectively, by Miss Moriarty, the Home Sister, and every course is followed by an examination. The annual report expresses the grateful thanks of the Council of the Nightingale Fund to Mr. Lunn; the

Matron, Miss Vincent; and to Miss Moriarty for the care and attention bestowed by them on the nurses and probationers. There have been 36 probationers in the Home during the year; of these 14 left before the end of the first twelve months, owing, in the opinion of the Council, "to a want of due appreciation on the part of the probationers of the advantage which service in parochial infirmaries holds out to nurses."

### LORD WINCHILSEA'S SCHEME.

It has been asked us on several occasions of what Lord Winchilsea's scheme for village nursing consisted. He has lately published a letter on the subject in which, being fully alive to the financial difficulty, he advocates the services of the trained nurse in cottage homes. He proposes that the parishioners should subscribe a small annual sum towards defraying the expenses attendant on employing the services of a "good nurse," whom he describes as being so great a blessing to the sick.

### SICKNESS AND SEWERAGE.

The borough of Stockport is gaining unenviable notoriety just now for its disastrously insanitary condition. There is much sickness in the town, at which no one can wonder who has read of the defective house drainage, and of new habitations erected over hollows "filled up with all sorts of rubbish and filth" after the natural sandy soil has been carried away. Naturally the death-rate is much too high, and it is likely to continue so whilst the teachings of sanitary science and all the laws of health are thus wilfully defied. It seems hard that the penalty of bad drains should have to be paid, not by those who lay them down, but by the innocent and ignorant occupiers of these houses.

### WELCOME NEWS FROM IRELAND.

"I don't think I should be afraid of the day work, but I should not like night duty," is quite a common remark when thoughtful unprofessional people discuss the different branches of nursing. By the time she has completed her training, every true nurse realises fully the great responsibilities and importance of this same "night duty." A patient, especially a weak and feverish one, soon discovers the blessing of having a regular trained nurse at his service through the dark hours which try him far more than any in the day. Therefore, the sick inmates may well rejoice over the new departure at the Mater Misercordiæ Hospital, Dublin, for the nuns have set a noble example by arranging to have trained night nurses and probationers in their institution for the future. This is as it should be, and doubtless other sisterhoods in Ireland and elsewhere will not be long in taking similar measure to secure skilled attendance for their sick charges. Under no other conditions is it possible for the doctor's orders and treatment to be accurately carried out.

### GAY DOINGS AT DINGWALL.

At the opening of the bazaar in aid of the funds of the Dingwall Nursing Association a very interesting speech was made by Lady Aberdeen. It touched upon the qualities and special tact needful in nurses selected specially to serve the sick poor in their own homes, and on the valuable practical instruction in sanitation which it is in the power of district nurses to disseminate. Other excellent speeches were made, all showing universal appreciation of the past and present work of trained nursing in Dingwall and the neighbourhood. The bazaar, which lasted two days, was held in the Masonic Hall, and the results of this admirably-managed fête should considerably augment the funds of a most deserving association.

### A REASONABLE COMPLAINT.

No one should resent a reasonable complaint. But those who regard the proportion of one nurse to three patients in any institution as inadequate should first turn their attention to rectifying a much worse state of affairs, such as, for instance, prevails in our workhouse infirmaries, and has been the case for some time at San Francisco, according to our information. In the County and City Hospital of that city but one nurse to a ward of thirty patients has been provided. Here is overwork with a vengeance, and if neglect of patients were not the result we should be much surprised.

### AUSTRALIA.

We hear not only of the present poverty of Australian hospitals, but also of excellent universal attempts being made to raise funds for these charitable institutions. The Misses Krakonski organised a bazaar in aid of the Children's Hospital at Melbourne, and the pupils of the North Melbourne High School recently arranged another to help forward the "Cot-Endowment Fund," in connection with the same hospital. We mentioned before that the sum of £3,000 had resulted from a "self-denial" week in Sydney, and now Melbourne is boasting of having raised twice as much money in spite of "bad times."

### A NURSING HOME IN MADRAS.

About six months ago Mrs. Nisbet opened a nursing home in Madras. The undertaking, although attended with some risk, appears to be on the high road to success. On the six months' accounts Mrs. Nisbet is able to show a good balance, nearly equalling the subscriptions, proving that the Home already is not very far from self-supporting. Her staff has consisted of two European and three Eurasian nurses. She has received a large number of applications for employment by certificated nurses. Encouraged by her success, she is now about to open a convalescent home for invalids.

### SHORT ITEMS.

The annual report of the Littleborough Sick Nurse Fund tells of 3,216 visits paid to 180 patients by Sister Lucie, who continues indefatigable in her labours. She has gained the approbation of her Committee and the warm affection of her poor patients.—Dr. Braidwood, of Halkirk, has received from his Sternster Nursing Class the gift of a fine reading lamp.—Miss Clara Streeting has been appointed nurse at the Norwich Infirmary.—The Newton Board of Guardians have appointed Miss Pyke as nurse at £30 per annum.—A successful course of lectures on "Home Nursing and Hygiene" have been given recently in Oxfordshire by Miss Lucy Scott, of the Ladies' Sanitary Association. They were greatly appreciated by large and attentive audiences, who enjoyed the pleasantly-given instruction of a qualified nurse. Miss Scott has undertaken to give a course of lectures on the same subjects in Shropshire in October.—The Grimsby Infectious Diseases Isolation Hospital has been specially reserved for cholera patients, and Miss Jackson, of the Grimsby Nursing Institute, is at the head of the nursing department. The steamer "Bradford" has been fitted as a Hospital ship, and is lying in the Humber, the nursing staff being under the charge of Miss Helen Norfolk, of the Grimsby Nursing Institute.

### The Truth about the London Hospital.

A TESTAMUR volunteered and signed by 246 old London Hospital workers is indeed a document which the Committee and Governors of the London Hospital must regard with profound satisfaction. The nurses are scattered far and wide, and few of them have anything either to lose or to gain by loyalty to their old training school. They are absolutely independent of it and removed from its authority, and therefore are quite free to join with any discontented persons in throwing insulting accusations at the walls within which they once worked. The present moment might certainly be seized by any old workers, for if it is easy "to hit a man when he is down," it is a thousand times simpler to hurl vile abuse against an impersonal institution. The man may rise up and hit back at his cowardly assailant, or be happy enough to possess friends who will afterwards bring an action for assault, so it behoves his enemy to be comparatively cautious. But when it is only an institution, albeit one of the best in the world, everybody can wreak his ill-temper and let off his superfluous humours against the huge and hard-worked establishment. But these women, the faithful "old workers," prefer showing that they possess the courage of their opinions, and they may well be congratulated on the simple and straightforward address handed in to the House Committee this week. It runs as follows:—

Gentlemen,—We have seen with much indignation the renewed attacks on the management of the London Hospital that have lately appeared in the *Pall Mall Gazette*. As old workers we seize this opportunity for offering an expression of our unfailing loyalty to the institution to which we owe so much. We should like to say how grateful we are in our daily work now for the teaching and experience we gained while at the London Hospital. The sound system of management, the discipline, the thorough training of nurses, and the interest we were taught to take in every advance towards a high standard of work have been invaluable to us all, but especially to those of us who now occupy important posts in the nursing world.

Many of us in the first years of Miss Lückes's work at the Hospital were privileged to help her in her unwearied efforts to raise the standard of nursing, and to improve the condition of nurses. We have rejoiced in the success which crowns her work, and are proud to be numbered amongst her nurses. This being the case, we wish to express to you how strongly we resent such malicious and unfounded imputations on the efficiency of the London Hospital as have lately appeared in the *Pall Mall Gazette*.

Whilst, however, we deeply deplore that such work should be harassed in the slightest degree, or made one whit more arduous than it must necessarily be, we have no fear for the ultimate result, chiefly because we have faith in our matron and in our hospital, and also because the traditions of that hospital are carried far and wide by an ever-increasing band of workers who are jealous of its reputation and united by the common bond of their allegiance.

We respectfully request that you will convey this expression of our loyal feeling for the London Hospital (its committee, medical staff, and our matron) to his Royal Highness the President, and to the Court of Governors, and will take such means to make it public as you may think desirable.—We have the honour to sign ourselves, gentlemen, your obedient nurses,

Signed by 246 OLD LONDON HOSPITAL WORKERS.

September 4th, 1893.

We also give a letter from present members of the nursing staff, which has been signed by 264 out of a total of 281, and from the latter figures we must substract six new probationers "on trial," who were not invited to give their names. The slip signed by Sister Buxton accompanied each copy of the letter. We need hardly remind our readers that Sister Buxton is not only the senior member of the nursing staff, but she was on it long before the hospital attained to its present dimensions, and years before the appointment of the present matron. She has, therefore, enjoyed greater opportunities than most people of judging of the development and progress which has taken place.

You are requested to see that each nurse and probationer in your ward reads this letter, and has the opportunity of signing it if she wishes to do so.

(Signed) SISTER BUXTON.

London Hospital, Whitechapel Road, E., September, 1893.

To the House Committee and Governors of the London Hospital.

Gentlemen,—After many discussions amongst us, we have decided respectfully to lay before you the united protest of the undersigned, your nursing staff, against the persecution to which our institution, our matron, and ourselves have been subjected during the last three years, and which has now broken out afresh in the *Pall Mall Gazette*.

In taking this step, we are quite aware that our accusers will not hesitate to declare, as on former occasions, that we have been coerced into doing so. Therefore, we feel it necessary to begin by stating that this action is entirely voluntary on our part.

We ourselves are well aware that nothing would be more repugnant to the authorities whom we serve than the bare idea of interfering with our freedom of thought or action, and it must surely be evident to all unprejudiced persons that if any desire to oppress us existed, it would be wholly impossible to make a large body of free and independent women get up and sign anything of this sort against their own honest convictions. We, therefore, wish this to be taken, in the first instance, as an indication that we decline to be intimidated by anyone in taking any action we think right.

Besides many petty and unfounded accusations, we are charged with culpable waste of hospital provisions; with allowing patients to suffer through our ignorance and neglect; and it is even said that we treat sick and suffering children with cruelty.

We entirely deny the truth of these statements, and claim that we are not less interested in the welfare of the patients than the medical staff themselves, whose well-known confidence in us is a great encouragement in our work, and from whom we all receive unfailing kindness and courtesy.

We resent the imputation that these evils exist, as much as the equally false statement that they arise from mismanagement, for if such had been the case, we should certainly have felt it our united and bounden duty to call the attention of the authorities to such a disgraceful state of things.

Surely no reasonable person can suppose that amongst such a large number of women, none were sufficiently constiencious to bring wrong-doing to light, even if they thereby ran the risk of damaging their own professional prospects, which our accusers assume would be the case.

We object to being represented to the public as a down-trodden spiritless body of weak, incapable, discontented women, thinking of nothing but our food, our times off duty, regarding it a trouble to attend to our patients, and unable to fight our own battles if the need arises.

What we wish to make clear is, that if grievances do exist, we are capable of laying our own case before our matron and the Committee without the intervention of outsiders.

It must be plain to everyone that those without any practical knowledge of hospital work are the least qualified to lay down the law concerning the supposed needs of nurses and patients, however much they may desire to pose as the friends of helpless nurses, and those who, with a mere smattering of knowledge gained by brief and unsuccessful experience here, have taken upon themselves to dictate to experienced workers, how wards are to be managed and patients are to be nursed, have at any rate succeeded in proving to us all how utterly unworthy they were of any permanent connection with us.

There is no injustice done to the nurses here equal to the cruel injustice done to our matron by the most unjust and personal attacks, which are constantly being made upon her by the same few persons, the truth of which, as you are well aware, is indignantly denied by a very large majority of past and present London Hospital sisters and nurses.

The sisters, specially, are well aware of the terrible strain under which the matron has carried on the work for the last three years, and they wish to bear testimony to the unswerving support she has always been to them, and without the certainty of which hospital discipline and training would have been absolutely impossible.

We are quite conscious that the Treasurer, the Chairman and the Committee, must know from their own personal observation, that what we have stated is and always has been the general feeling on these matters throughout the hospital, and as we are not a set of weak-minded women, all the persistent efforts made to shake our confidence have been in vain. But we should also like the public to recognise, that whereas the attacks come mainly from outsiders and disappointed probationers, whose views are probably not unbiassed by their own failures, the refutation comes from tried and experienced workers, many of whom have been in your service a number of years, and who have given evidence that their desire is to show zeal and devotion in their chosen work, and to do credit to the training they have received.

We have decided amongst ourselves that the time has come for us to make this formal and united protest against these mischievous attacks, and to express our firm unshaken loyalty to the House Committee, the medical staff, and our matron.

It is our earnest request that you will kindly have this manifesto of the nursing staff of the London Hospital made known to the President and the Governors, that they, receiving this assurance of our perfect contentment with existing arrangements, will do their utmost to put a final stop to the constant interference of our so-called friends, the result of which is only to cause needless annoyance to all concerned.

Finally, we venture to express our wish that as these malicious charges against us have been so industriously set forth by our accusers in the public press, you will kindly give equal publicity in the same direction to this voluntary statement by those who have unquestionable right to form and express their judgment on the questions raised.

### The Work of Deaconesses in Germany.

*Prepared for the International Congress of Nurses.*

The following *résumé* of the work of deaconesses in Germany has been written to accompany the book published in Kaiserswerth, in 1886, as a memorial volume for the fiftieth anniversary of the founding of the Kaiserswerth institutions :—

The beginnings of the work of deaconesses in modern times were small and unsightly, like the mustard seed. To-day their cause is bound up heart and soul with the Protestant Church in Germany, and deeply rooted in the life of our people. In the place of one mother-house at Kaiserswerth, begun fifty years ago with one deaconess, there are now sixty vast establishments with six thousand deaconesses in successful operation. The host of these volunteers against all kind of human need and misery are upon duty by day and night everywhere, from under the palms of Egypt unto the snow and ice of Lapland, from the sacred grounds of Zion and Lebanon unto the scene of the latest beginnings of Christian culture in the Far West of America. The man who gave the impulse to an efficient renewal of the ancient office of the women helpers, a renewal adapted in its form to the wants of to-day, was Theodor Fliedner, a clergyman of the Protestant Church. When, in 1822, in his twenty-third year of age, he was appointed minister to the Evangelical community of the small town of Kaiserswerth on the Rhine. The modest youth arrived in his parish on foot in order to spare to his poor parishioners the cost of the usual welcome. Poverty was Fliedner's lot through all his life, and it was that of his parishioners, but their very destitution showed the way to him from which he brought back the stimulus to the great work of his life. When Fliedner had been for a few weeks in Kaiserswerth a rich merchant who had lent 500 thalers for the building of the Evangelical church, became bankrupt, consequently the church had to be sold. Two other places were at the same time offered the young pastor. But he could not bear to witness the destruction of his parish. His faithfulness led him to travel about through the Rhine Provinces, through Holland and England, to raise funds to save his church. He actually brought home the desired sum, but, more than this, he had gained the knowledge of great charitable enterprises of other nations, especially that of the care for the prisoners. Soon after his return he began trying to work in the same field. Journeys to this purpose caused his acquaintance with the Rhenish pastor, Franz Klonne, who for a long time had urged on the public thought a revival of the ancient ministry of the deaconesses. Fliedner had actually found women helpers officiating in the parishes of the Hollandish Mennonites. He resolves to found an asylum for discharged women prisoners, and to apply to Christian womanhood for support of his work. In 1833 the first woman prisoner, soon afterwards the first deaconess, arrived in Kaiserswerth. Only a very small summer-house, twelve feet square, in his own garden, could as yet be obtained for the two. The book, by J. Disselhof, will tell you minutely how wonderfully the grain of mustard seed became an overshadowing tree.

I here restrict myself to a short description of the present state of the cause, which cause, exclusively by Fieldner's never relenting energy in persuading, working, collecting, leading, gained a vitality that will never more give way. In 1836 it was possible to buy a house, a very stately building then in the eyes of Fliedner. It is true that the price of 2,300 thalers could only be raised as a loan by the good pastor's indefatigable entreaties. But the sympathy with the work increased, and so did the establishment. The activity of the deaconesses from the care for prisoners soon extended to the nursing of any kind for the sick and the needy. Christian education of children, as well as that of their teachers, the care for the insane and the Magdalens became new branches of the work, which, indeed, tended to alleviate every sort of human misery so far as woman's ministry might lend a helping hand. To-day we find in Kaiserswerth a great hospital, a fine church, and a college, a home for old and invalid deaconesses, a workmen's house, an orphan asylum, an asylum for prisoners; we find kindergartens, a seminary, a library, preparatory schools for teachers and helpers, bathing houses, and the cottages of the various officers in the vast establishment. Within few years after its foundation the mother house could open new "stations" all over the Rhine provinces. The number of these new places of work is to day 107 in Rhineland and 36 in Westphalia. Filial houses were built in other provinces and even in distant countries. It was granted to Fliedner to witness this wonderful development. His blessed life ended October 4th, 1864. He had been married twice, and each of his wives was a faithful helper in his work and a model to other deaconnesses.

Let me now review in short the statutes for the order of the deaconesses. You will find ample and interesting information on the subject in the book by Disselhof. The thirty-fifth year is the utmost term for the admission of a "sister." She has to begin with a preparation of some years' studying and working in one of the headquarters. The preparation is fixed longer or shorter acccording to character, capacities, and education of the individual. It comprises the practice of the most humble household work as well as the higher instruction for all the above mentioned disciples. If at the end of an appointed time the novice is judged fit by the women superintendents of her house to be a deaconess, she is consecrated to the ministry in a religious ceremony. The relation to her family remains undisturbed, as well as her disposition and administration of her private fortune. At any time the sisters are in close connection with their mother house, the goverment of which is in the hands of a lady superintendent (Frau Oberin). The house appoints the position and mission of each of its members, and takes care of them. They are at liberty to marry and return to their parents if the latter want and require their nursing.

Until 1884 more than 60 houses of deaconesses with nearly 6,000 sisters and 1,750 outside working places had opened.

I shall finally name the principal among the establishments.

The first of them was the Elizabeth Hospital in Berlin, founded under the protection of Queen Elizabeth of Prussia, in 1837. The number of its deaconesses to day is 101.

A hospital in Paris followed in 1841. It was re-established in 1867, once more in 1874, with to-day 15 sisters. The hospital in Strassburg, 1842, has to-day 165 sisters.

St. Loup in 1842, 54 sisters; Dresden, 1844, 218 sisters; Utrecht in 1884, 64 sisters; Berne, 1845, 210 sisters; Berlin, "Bethanien," 1847, 223 sister, Stockholm, 1849, 136 sisters; Pittsburg, now Rochester, U.S., in 1849, 18 sisters; Breslau, 1850, 175 sisters; Konigsberg, 1850, 204 sisters; Stettin, 1850, 32 sisters; Ludwigslust, 1850, 140 sisters; Karlsruhe, 1851, 89 sisters; Riehen, near Baset, 1852, 174 sisters; Neuendettelsan, in Bavaria, 1854, 228 sisters; Stuttgart, 1854, 286 sisters; Augsburg, 1855, 63 sisters; Halle on the Saale, 1857, 70 sisters; Darmstadt, 1858, 135 sisters; Zurich, 1858, 80 sisters; St. Petersburg, 1859, 34 sisters; Speier, 1859, 70 sisters; Kraschnitz, in Silesia, 1860, 74 sisters; Hanover, 1860, 189 sisters; Hamburg, "Bethesda," 1860, 27 sisters; London (in Hyde Park), 1861, 14 sisters; Danzig, 1862, 93 sisters; Kopenhagen, 1863, 115 sisters; Tregsa, now in Kassel, 1864, 34 sisters; Haag, in Holland, 1865, 35 sisters; Milan, in Kurland, 1865, 14 sisters; Posen, 1865, 66 sisters; Budapest, 1866, 10 sisters; Frankenstein, in Silesia, 1866, 121 sisters; Riga, in Livland, 1866, 10 sisters; Berlin, "Lazarus Hospital," 1867, 43 sisters; London (Tottenham), 1867, 39 sisters; Reval, in Esthland, 1867, 18 sisters; Heling fors, Findland, 1867, 12 sisters; Altona, Holstein, 1867, 58 sisters; Bremen, 1868, 23 sisters; Christiana, 1868, 172 sisters; Wyburg, 1869, 5 sisters; Bielefeld, 1869, 352 sisters; New Torney, near Stettin, 1869, 150 sisters; Flensburg, 1874, 76 sisters; Berlin, "Paul Gerhardt House," 1876, 55 sisters; Sarata, in South Russia, 1867, 21 sisters; Nowawes, near Potsdam; Gallneukirchen, in Austria; "Selim," near Stettin; "Bethlehem," in Hamburg; Arnheim and Philadelphia, U.S.

### Cottage Nurses.

(*From a Correspondent.*)

In an interesting article which appeared lately in The Hospital on "Associations for the Supply of Cottage Nurses," a scheme was described which is to be set on foot in Dorset. It is proposed to amplify and improve the Holt-Ockley system in this, all-important particular each cottage nurse is to be *under trained professional supervision.*

It is greatly to be hoped that the promoters of the original Holt-Ockley Association in Surrey will also see the immense advantage of placing trained superintendents over their nurses; it is quite impossible that the ladies who now undertake to oversee the cottage nurses should be able either to judge of their capacity—or what is equally important—to give them further training.

Some time ago I had an opportunity of gaining some knowledge of the Holt-Ockley Benefit Nursing Association, and of comparing it—if a comparison can indeed be made—with the work of the Metropolitan and National Nursing Association in London. I need scarcely say that my firm belief in the importance of supplying educated and fully-trained nurses for the sick poor was confirmed; and I also believe that so much would be gained by placing the cottage nurses under skilled supervision, that it can only be a question of time before this is accomplished.

I know, of course, that the objection ordinarily made to this proposition is that of the greater expense involved. This at the present time, when in other parts of England first-rate skilled nursing is rightly considered essential for the sick poor, seems scarcely worth consideration; and I believe that people could easily be convinced, first, that to supply third-rate nursing is no true economy; secondly, that by supplementing the work of, the cottage nurses by means of fully-trained nurses better work would be done; and, thirdly, that if this alteration were once established more cases could be nursed.

It sometimes happens under the present Holt-Ockley system that no cottage nurse is available for a fresh case, the only alternative then being either to take a nurse from a patient who is recovering, or to keep the new case waiting till a nurse is disengaged. It is obvious that a superintendent nurse who could pay daily visits would here be of very great value.

In many cases, also, a nurse is not wanted by the cottage people to live altogether in the house; in fact, unless the mother of the family is ill they generally prefer daily visits, and probably the larger part of the work of Surrey cottage nurses consists in the care of women during confinement, at which time it is doubtless a boon to have a reliable woman in the house to look after the children.

But this, of course, means additional expense, and it is partly for this reason that the cottage nurses are rarely called in to nurse either men or children, the mother of the family thinking, perhaps with some reason, that she can do it just as well. The doctors have also discovered that the standard of training is too low for them to be able to expect much in the way of real nursing, and, therefore, much ground remains uncovered that would be occupied by a well-trained district nurse.

Although the cottage nurses professedly pay daily visits this comes to very little, as they are generally out at cases, and are not available for such work as daily surgical dressings. Here also a well-trained superintendent or district nurse could supplement their work. She would also be directly responsible to the doctors for the nurses working under her. She would personally superintend the nursing of each case, and be able to counsel and instruct the cottage nurse in any difficulty, perhaps keeping the charting of temperature, pulse, and respiration and details of skilled nursing in her own hands, but always bearing in mind the necessity of training those under her as far as possible; it is a mistake to suppose that any woman really interested in her work would not value the opportunity thus given her of learning more.

A superintendent would also be present at all operations. I knew of a case where tracheotomy was performed, and the cottage nurse left in charge had absolutely no former experience of a case of the kind.

It might be necessary to have several fully-trained nurses to undertake the daily visits, as well as a superintendent, and most likely a pony cart would be found a great help. I also believe that another name than that of "nurse" should be found for the so-called cottage nurses; their training or testing is too insufficient to give them any right to such a title, which at present leads to confusion, and lowers the standard as to what a nurse should be, at least amongst the poor.

Perhaps, however, there is no improvement which would be more certain than that in the direction of hygiene. Cottage nurses, belonging to the same class as the patients, have neither the knowledge nor the authority to enforce sanitary measures. Surely the whole question is worth reconsideration. It has been proved that education and refinement are no disadvantages, but rather a gain, to nurses, whether their work lies in the country or in towns, and those most competent to judge urge that the poor need the best nursing. So much knowledge and training are required to meet emergencies which may arise even in nursing the simplest cases; so much absolute certainty, which experience alone can give, is needed if the nursing is to be both intelligent and trustworthy; so much tact and courtesy are wanted if the standard of nursing and living are really to be raised amongst the poor; and of this I am sure, no better work can be done for them than by supplying a class of nurses sufficiently educated to carry out fully the doctor's orders, and at the same time to raise the moral tone amongst their patients.

### Everybody's Opinion.

[Correspondence on all subjects is invited, but we cannot in any way be responsible for the opinions expressed by our correspondents. No communications can be entertained if the name and address of the correspondent is not given, or unless one side of the paper only be written on.]

### NURSES' UNIFORM.

"One who Wears a Fringe" writes: May I say a few words on the important questions, viz., a nurse's dress and appearance, at present being discussed in the pages of your valuable paper. Is it not every true woman's duty to dress as prettily and to look as charming as possible, and why should nurses be an exception to this rule? A fringe if kept tidy is a great improvement to many a plain-featured woman like myself, and it is hard upon us that for so doing we should be classed as "flirts" or frivolous "husband hunters." As to the question of out-of-doors uniform, a tired nurse, longing to make the most of her time outside does not care to waste half of it changing into every-day costume with all its accompanying fal-lals, fastenings, and trimmings which Mrs. Grundy considers necessary for a respectable appearance abroad. If urged on the plea of infection, I can only say that changing the dress alone will not prevent this, as the underskirts and clothing are just as apt to carry the germs of disease. If the out-of-doors uniform is granted, then, while keeping it as simple as possible, let it also be becoming. Working in low parts of town, I have also often found my uniform a great protection as well as comfort. One is sorry and ashamed that an unprofessional correspondent has such a low opinion of the majority of noble women working in our midst. Surely few, and let us hope an ever decreasing minority, are actuated by such ignoble motives as supposed in joining our ranks.

Nurse Agnes writes: Respecting the paper No. III., by an "Unprofessional Correspondent" in The Hospital of September 2nd, allow me to say, 1, that, thanks to the nurses' uniform, I am able to save from £20 to £25 a year, as my savings' bank book can show. All of this would go on hats, jackets, boas, muffs, and a dozen other things did I adopt mufti and try to keep with the fashions. 2. "Unprofessional Correspondent" says the veil could not be placed over the face. I beg to differ. This year I have had fifteen weeks of seaside nursing, a windy, sandy place, when the wind blew the sand into my face—I just pinned my gossamer veil across and saved my eyes, and was able to grin at those poor souls who tried to hold up umbrellas, or blink behind their tiny, thin veils. 3, Again, why should not we put on clean cuffs to walk in, instead of those worn all day, and splashed, perchance, with medicine, antiseptics, &c.? 4. Again, there is another and very serious reason for the non-extinction of outdoor uniform, as no one knows who she may sit next, in a public conveyance or theatre, and if he or she sees nurses' uniform, they may take another seat and steer clear of infection. In a hundred ways I find my uniform a blessing and a comfort; it cleared the way for me in a dense crowd the other night to bring out into the fresh air a fainting young woman who was nearly crammed and pressed to death. "Make way for the nurse," was the open sesame, thanks to uniform.

### WHO SUPPLIES THE DRUGS?

"M. F." writes: I should be glad to know whether it is the practice throughout England that the parish doctor, receiving a small salary, should be expected out of that to provide all necessary drugs for his pauper patients. In a district known to me where this practice prevails with a population of about 17,000, this doctor, I believe, only receives some £60 a year as his salary, and one cannot but see that such an arrangement is likely to produce an antagonism between his immediate pecuniary interests and what, apart from these, he might consider to be the medical requirements of his patients. Without imputing that any neglect, in fact, results, it seems unfortunate that any avoidable temptation should exist threatening the well-being of pauper invalids.

### TEA DRINKING.

"R. S." writes: As a sincere lover of tea as tea should be, I would give comfort to my fellow "tea drunkards" in assuring them that so long as they respect Sir Andrew Clarke's condemnation of Indian teas they need be under no apprehensions of ill effects from indulging in the China product. Palates are no doubt vitiated, as you put it, by the coarse Indian teas, but this depraved taste will soon pass away, and the tea-drinker revert to the pure tea-flavoured beverage as of old, and take care to avoid the hyper-tannated teas now being condemned by doctors on all sides.

### AN ANSWER TO AN UNPROFESSIONAL CORRESPONDENT.

A Hospital Nurse writes to us as follows, signing herself "Justice": I could not help thinking, as I read the opinions of the "Unprofessional Correspondent," that it would have been better had she kept them to herself. Which is it she deplores most in a nurse? The terrible fact that she sometimes marries a doctor, or the adoption of out-door uniform? The nurse is often compelled (regardless of her own wishes) tc wear whatever the authorities of her particular hospital or institution ordain, and I think she should awaken pity, rather than envy or malice, in any true woman. Does not common sense show that anyone, especially in hot weather, would rather not be clad in serge dress, heavy cloak, and bonnet with veil and strings? If outsiders only knew how gladly the nurse would exchange these appendages for pretty, dainty dresses and shady hats—yes, and even the much maligned white collars and cuffs would be willingly sacrificed. Is it not just possible that the style of dress of you "mondaines" (as you so appropriately term yourselves) might be infinitely more "attractively aggressive"? Why a girl should be expected to give up every pleasure in life is difficult of comprehension. It is the girl or woman who keeps her interest in tennis and occasionally visits the theatre who often makes the best nurse. She is naturally bright, sympathetic, broad-minded, and able to define fairly the vast difference between pleasure and pain. People are so apt to become mere machines in hospitals. There is little time to read the papers or to discuss events in the outside world. Pain and suffering are always present, and it is quite possible for even the most sensitive nature to become blunted and hardened, without some forcible contrast being brought into their lives. One thing I quite agree with, and that is that talking "shop" should most certainly be kept for the wards. Nurses who wear white aprons when they go for a walk would often say that they had never thought of omitting what is part of their ordinary attire. Can a simple white apron be considered attractive in any way? Poor nurses, the absolute cleanliness of their clothes is the only luxury about them! There is no variety, nor discussion, or choice of colour, &c.; just the same the whole year round; yet still room is found for complaints and for the "absolute abolishment of white adornments." Are sack-cloth and ashes desirable, and should nurses take a vow of celibacy when they start on their career? What did most people think when they read that "because nurses receive but a small salary they belonged to a class of persons who were too ignorant or silly to earn a living otherwise"? On the great calling of nursing should be bestowed the very greatest intelligence, care, and thought, and how could it be followed by people incapable of getting a living in any other way? There are, of course, still girls who regard marriage as the one end of nursing life, but if that be their aim why do they not stay in the world of society, where they could find so many more opportunities of talking to doctors, students, or other men than they get in their twelve or fourteen hours of often weary duty? A nurse's life is arduous, and by no means all girls should be advised to adopt it. To those who cannot apply practically the grand doctrine "Not only with our lips, but in our lives," the life must be a weariness to themselves and a discomfort to their patients. There are good and bad of every class; but assuredly there are hundreds of willing, self-sacrificing girls to be found in our hospitals to-day who live up to this ideal, and yet bring plenty of honest good nature and mirth into their work outside and inside their institutions.

### Notes from Philadelphia.

(By Our Own Correspondent.)

### UNIVERSITY HOSPITAL, PHILADELPHIA.

There is very little of interest transpiring at present in this hospital. Clinics ceasing about June 15th, staff, officers, nurses, and employés take advantage of the fact and go off on vacations for a longer or shorter period as their position or their needs warrant.

The principal improvement that is occupying the minds of the authorities at this time is the new laundry which will be fitted up in the most approved style for laundry work, also with sterilisers for infected clothing and bedding, and with a sun room for airing beds that are not in every day use. Another great advantage from the building of the laundry will be an upper storey for the accommodation of the female employés, who have heretofore been lodged in rooms over the wards, which rooms are very much needed for the purposes for which they were originally intended. It will also take the housekeeper and head laundress out of the building, and the additional rooms will enable a scheme to be carried out to introduce trained nurses into various departments, over which they will exercise a general oversight.

A trained nurse is already working in the Superintendent's office to give her some idea of the general management of hospitals, so that if she has a desire, and an opportunity presents itself, to take charge of a small hospital, she will have some idea of the financial management which simply training for a nurse never gives. The trained nurse will next pass through the housekeeping and other departments, so that the authorities may be able with some degree of confidence to recommend her for any position she seeks or desires to occupy. Lack of accommodation has prevented these plans from being heretofore fully carried out, as although the hospital possesses a very pretty Home for its nurses, the staff is steadily outgrowing the accommodation it affords.

In my next I will tell you something about the other hospitals in this city which have training schools which have many interesting facts connected with them.

### News from Australia.

(By Our Special Correspondent.)

In spite of the extreme commercial depression at present existing in Melbourne (Victoria) a new hospital will shortly be opened. The nursing sisterhood of St. Vincent de Paul have for many years had a general hospital at Sydney (N.S.W.). and a branch house has been established in Melbourne. A terrace of three houses, each about ten rooms, has been rented in Victoria Parade, a very wide street, with trees and grass down the centre. Two of these houses are required for official purposes and the accommodation of the sisters. The third will contain about 30 beds. As is the practice of the head establishment at Sydney, accommodation for paying patients will be provided, and whilst no one who applies for admission will be turned away, those who are able will be expected to contribute something, however small a sum, towards the funds of the institution. A ball in aid of its funds was given in May, and resulted in a handsomes um. The opening of the hospital has been delayed by necessary repairs and alterations, but it is thought that these will be sufficiently advanced by September to admit of commencing work. The first St. Vincent de Paul hospital is likely to be much appreciated by the Roman Catholic body in Melbourne.

Part of the Alfred General Hospital, Melbourne (Victoria), has been closed. This has been necessitated by want of funds, the decrease in public subscriptions and Government grant being a consequence of the commercial crisis through which the colony is passing. The committee of the Austin Hospital for Incurables have had to close the consumptive wing. Upwards of 50 applications for beds now lie for consideration, and yet everyday the probability that some of the general wards of that institution will also have to be closed draws nearer.

The annual election of six members of the committee of the Melbourne Hospital took place on Friday, July 28th, with the result that only two of the retiring members were re-elected. Mr. George Godfrey and Mr. Robert Showers were the old members so honoured, whilst the new men were Mr. J. McDonald, Mr. J. J. Brenan, Mr. S. P. Akehurst, and Mr. Theo. Fink. Considerable dissatisfaction with the mode of election exists, not only amongst the subscribers, but also in the minds of members of the committee.

### For Reading to the Sick.

### PRAYER—II.

In the far East, and especially in India, the inhabitants think much about their religion, and are terribly afraid if they neglect to pray to the ugly idols they worship, that their gods will punish them. To prevent this, and to give themselves as little trouble as possible, they have invented praying machines into which they put the prayers they want granted, while a little windmill at the top keeps them turning round and round, and all the time they are in circulation these poor ignorant people fondly imagine they are doing the best for themselves, body and soul. They have another short road also to the same end. They choose the name of their favourite god. "Siva," for instance, is very popular, because they imagine him to be mighty and powerful, and they repeat "Siva!" "Siva!" "Siva!" as fast as they can for fifty, one hundred, or perhaps several hundred times, for they imagine that the longer they keep on, the greater will be their blessings.

Now it was against such superstitions as these that our Lord warned us in His Sermon on the Mount. "When ye pray," said He, "use not vain repetitions as the heathen do, for they think they shall be heard for their much speaking," but unfortunately we are often, too, like them. When we do not put our hearts into our prayers, we resemble the "windmills," the words flutter over our lips, while all the time our thoughts are wandering to the last new dress we bought, or to the coveted new book, or to the proposed pleasure trip which dangles before our eyes with a brilliancy which can never be realised. Then again, when we have started with the best intentions, we are often ashamed to find ourselves with the holiest names on our tongues while our minds are far away, and we are no more praying than those who say "Siva!" "Siva!" This inattention in prayer is a great trial to good people who make many efforts to overcome it. A devout position is a great help to fixing our thoughts on the matter in hand, such as kneeling, or if we are on a sick bed lying very still, closing our eyes, and clasping our hands in supplication. True devotion is, however, rooted in charity, that is, love to God aud love to our neighbour, both of which we show in the words "Our Father." "*Our* Father," we say, "Give *us* this day *our* daily bread"; "Forgive *us our* trespasses"; "Lead *us* not into temptation"; "Deliver *us* from evil." Thus we join with all mankind in asking unselfishly that our Creator, our King, our Father, will supply *all* His children with grace, and food, and protection through life, and deliver us from evil for eternity.

Mrs. Furley Smith is well-known to many of our readers as a valued contributor to The Hospital. The following announcement will, therefore, be read with interest: "Smith.—At St. Peter's Lodge, Uddington, on 31st August, the wife of James Smith, LL.D., Barrister-at-Law, H.M. Inspector of Schools; a son."

### The Muses' Looking Glass.

### WOMANLINESS.

Charles Kingsley, on being asked to write some answers to certain questions as to what he most admired and what he most disliked, wrote in answer to what he most admired in a woman, "Womanliness."

In these days, perhaps, it may not be altogether fruitless to call to mind that there is such a quality, for in truth one has cause on looking round to fear lest it may be in danger of "dropping out." Perhaps we shall best understand what it *is* by first saying what it is *not*. It is not then mere femininity; it is not merely being "ladylike"; it is not merely being gentle and tender; it is not merely being refined and modest; but it is all these and a great deal more besides. Womanliness may be likened to the sunlight, which is composed of an infinity of rays, and yet appears only as one bright, beautiful whole. We do not realise this very often until a passing cloud overshadows some of these rays, and we feel at once a sense of chill and discomfort, and find the beautiful whole is broken up, and only a part of its warmth and brightness has come to cheer us. So it is with some women. They appear for a time all that is admirable, until some test arises by which the grace of perfect womanliness is proved to be wanting.

Womanliness does not necessarily presuppose an absence of the more powerful intellectual development which until recent years has been regarded as the essential property of man. A most womanly woman may have exceptional mental power and capacity, but her womanliness will so direct that power that it will be exercised in those things and for those purposes for which a woman rather than a man is eminently fitted.

We are not now calling in question the desirability of professions and employments being equally open to women as to men. If women are naturally fitted for taking certain positions which have hitherto been considered as belonging by right to the stronger sex, by all means let them have the means of competing for them. The not doing so will not make them other than they are, and there are some no doubt fitted by nature and characteristics to shine more and do more good in their generation in the less feminine pursuits of life, than they would ever achieve in the sphere that is called their own.

We are not, therefore, taking the case of those women who are competing with the higher order of men, but rather of the vast number who, inflamed by the excitement consequent upon the greater liberty now accorded to women as a class, show their appreciation of it by imitating not manliness, but masculinity; and with their close-cropped hair, tight-fitting clothing, and general appearance would seem to indicate a desire to imitate inferior manhood, rather than to fit themselves intellectually to compete with the greater brain power supposed to belong to the other sex.

All undue suppression generally ends in the madness of revolt, and before the emancipated can reap the benefit of the emancipation a season of wild extravagance has to be passed through. Such it would appear is the case at this time, and the grace of womanliness is endangered thereby.

In the greater liberty of thought and action which characterises the age in which we live, there is one essential quality which is, perhaps, more endangered than any other, and that is reverence. We see the absence of it in the children first—in the tone of equality which they take with their elders. The old familiar titles of "Father," "Mother," "Aunt," are now replaced by nicknames and an assumption of familiarity which, while it saps the essence of all reverence, love, and admiration, does not increase the happiness of the children or their superiors. To be treated like a lap-dog, and petted and fondled familiarly does not help a mother in her highest duty and responsibility, that of training and guiding her child by the light, often dearly bought, of a larger experience, to become a better and wiser woman than herself. And the child loses in the process the advantage, during its immature years, of that sense of a guiding and restraining power which is so great an assistance to the young. There is no doubt that the greater confidence existing between parents and children in the present day is an enormous improvement on the fear and formality which marked the relation of the two in a past generation, but we want the just medium in this as in other things. There is a dignity belonging to every relation of life, and when that dignity is lost the beauty of any such relation goes with it.

A "Holy of Holies" of some kind is needed to bring out qualities which are essential in a womanly woman, and the danger of the present time in this respect would seem to be that by doing away with "superstitions," so called, we are eliminating from the education of the women of the future an essential element of their very character as women. A woman without reverence is like a picture without shadow, and, although this may not appear at first, it will be found as time goes on that no after-training will give the softening influence that has been lost during the earlier years.

Few will, we imagine, be prepared to say that a woman's influence is unimportant in its effect upon the men with whom she may be brought in contact, but this is mainly through her grace of womanhood, and where this is deteriorated the beneficient action will be neutralized; therefore, to endanger in any way the womanliness of our women is to rob the future of one of its most powerful agents of regeneration.

### Appointments.

[It is requested that successful candidates will send a copy of their applications and testimonials, with date of election, to The Editor, The Lodge, Porchester Square, W.]

Mildenhall Cottage Hospital.—Miss S. M. Marsters has been appointed Matron of this hospital. She was trained and afterwards worked for some time at the Norfolk and Norwich Hospital. We cordially wish her success in her new work.

Beverley Cottage Hospital and Dispensary.—Miss H. E. Bland has been appointed Matron of this hospital. She was trained at the Sheffield General Infirmary in connection with the Sheffield Nursing Institute to which she was afterwards attached for two years. Subsequently Miss Bland joined the Huddersfield Institution, then became Sister at the Sheffield General Infirmary, and takes many good wishes with her.

### Minor Appointments.

St. William's Hospital, Rochester.—Mrs. Fletcher, a former Matron, has been elected to the same post at this hospital, her husband being at the same time appointed to the office of Curator. They previously held similar posts at the North Bierley Joint Hospital.

Norfolk and Norwich County Asylum.—Miss Laura Shulon has been appointed Assistant Matron of this institution. Miss Shulon was trained at St. Mary Abbots Infirmary, Kensington, and has since held posts at the Kensington and Chelsea Schools, at the Hanover Institute, and the South-Eastern Fever Hospital, New Cross.

### Notes and Queries.

### Queries.

(186) *District Nurse.*—How can I obtain a certificate for monthly nursing?—*Certificate.*

(187) *Cancer.*—How can I get a patient admitted into a cancer hospital free?—*Nurse Fidelis.*

(188) *Dispensing.*—Where can I learn dispensing?—*Mrs. A.*

(189) *Sulphuring.*—I want information as to sulphuring rooms.—*Nurse A.*

### Answers.

(186) *District Nurse (Certificate).*—At a lying-in hospital. If you want the L.O.S. you had better write for particulars to the Secretary, London Obstetrical Society, Hanover Square.

(187) *Cancer (Nurse Fidelis).*—Write to Miss Petrie, Home for Incurables, 50, Osnaburgh Street, London.

(188) *Dispensing (Mrs. A.)*—See chapter on Dispensing in "Burdett's Hospital Annual," published by the Scientific Press, 428, Strand.

(189) *Sulphuring (Nurse A).*—Instructions for sulphuring a room were given fully in one of the Sanitation articles recently published in The Hospital.

### The Book World for Women and Nurses.

[We invite Correspondence, Criticism, Enquiries, and Notes on Books likely to interest Women and Nurses. Address, Editor, THE HOSPITAL (Nurses' Book World), 428, Strand, W.C.]

QUIXOTE, THE WEAVER. By C. G. FURLEY SMITH. (Hurst and Blackett, Great Marlborough Street, S.W.)

This very interesting story tells how one Hugh Boswell, linen manufacturer, at Queenshope, a small Scotch town, offends his fellow townsfolk by excess of philanthropy. He has inherited a large sum of ready money, and this he devotes to rebuilding his factory and equipping it with every modern improvement. This makes him unpopular with his friends and his mill hands—the latter resent having to learn the use of new machinery, even though it in no way lowers their wage, and the former fear that they also may be expected to make costly improvements for the comfort and health's sake of their employées. Boswell's comprehension of his responsibility is much the same as the present German Emperor's. He desires to be a "benevolent despot," and such typical Scotch radicals as Peter Ranken and Jamie Macnair resent the library, the night-schools, and the concerts, which they consider but an attempt of Boswell's to "soothe his conscience by gi'ein us back as charity a sma' portion o' what's oors by rights, the money that we work for and he gets." Lindesay Lorimer, the lady whom Boswell would make his wife, but who decides otherwise, is a truly noble girl. She is evidently the favourite of the authoress; into her mouth are put all the sayings, arch, provoking, and practical, poetic, just and generous, which we can fancy the authoress would have wished herself to utter under the circumstances of the tale. There are many other character portraits of interest in "Quixote, the Weaver," but they are too numerous, and the plot altogether too involved, to attempt to give either in a limited space. To sum up our opinion, we might say that the character drawing is infinitely better than the plot, and the dialogue of the weavers and their friends far more naturally rendered than that of their masters. These latter are a little apt to speechify to each other and to soliloquise like veritable Hamlets when alone. We have criticised the book at length, because it is a first book and shows very great promise. If Mrs. Smith will give us more stories of characters she has studied from the life and omit such as are only conceived with a view to certain situations or as mouthpieces for ethical remarks, the reading public, and especially the jaded critic, will look forward to them eagerly and read and review them with gratitude.

THE MEDICINE LADY. By L. T. MEADE. (Cassell and Co.)

"The Medicine Lady" is a courageous attempt to combine hack writing with artistic merit; the result is both unpleasant and boring. Instead of thinking out an interesting plot and characters whose logical development shall interest both the author and his readers, we feel instinctively the hampering conditions under which the story must have been planned. We should opine, firstly, that the tale was written to order, probably as a serial required to contain a fixed number of chapters. Secondly, we feel that the "tone" of a magazine has had to be considered, Girls, we should say, had to be catered for, and very middle-classy, high-schooly girls, moreover. A "purpose" too, seems to have been re-

**THE BOOK WORLD FOR WOMEN AND NURSES**—*continued.*

quired. All these desiderata have received due attention. The Medicine Lady is an undisciplined, pert girl who has to learn through bitter experience that an unruly spirit brings trouble on others as well as itself (here we have the "purpose.") Dr. Koch's theory provides the "serious interest," and the every day sayings and doings of many persons of incredible vulgarity are supposed to supply "the comic relief." Thus all the presupposed conditions are fulfilled, and the result may be "good business," but is certainly very bad literature, and, we venture to hint, very bad morality too. There is far too much scribbling of this sort. It is so easily done that every schoolgirl thinks she could write as well herself, and most of them can. Thus we have innumerable misses wasting their time by writing, and the time of others by reading badly composed sentimental tales, containing not one original idea. The earth is over-cumbered with such things. Let no one write stories unless he or she have something really to say—something that they have not read elsewhere.

"The Princess's Private Secretary." Translated from the Italian of A. G. Barrili by His Honour Judge Stephen. (Digby, Long, and Co.)

"The Princess's Private Secretary" takes a long time to tell a small commonplace story in the language familiarised to us by our early French exercises; nowhere else is it to be met. Judge Stephen has preferred "literal" to "literary" merit, which may be an injustice to the author, as it is impossible to guess at his "style" from this undiluted dictionary version. Though this excuse granted, the construction of the book inclines one to think that Italian authors are still far behind the times. We see no reason for cumbering paper with such unoriginal matter. The author has nothing to tell, and tells it badly; the plot is feeble and the characters mere marionettes. Though the personages are all Italians but one, they might quite as easily have been invented by any untravelled Englishman who had read here and there of Italian customs, and had interpolated dull digressions having no manner of bearing on the tale. The nearest approaches to thrilling situations are blocked, and one's feeble interest allowed to wander elsewhere by tedious disquisitions on Bismarck, or the exact shade of meaning to be attributed to certain Italian political writers. The slight thread of story on to which these are hung tells of a young man with the stereotyped attributes of the *jeune premier*. Handsome, generous, brave, almost a genius, he is educated into a lawyer, and his father dies before he has time to acquire a practice. Lucio must find means to support his mother and five brothers and sisters, so he becomes private secretary to an elderly but flighty Princess, who is jealous of her stepdaughter's youth contrasting with her own *passé* charms. It is unnecessary to detail the sordid schemes of these great people, which become far more complicated when the handsome secretary comes to dwell among them. Despite his youth and inexperience, he acts throughout with the wariest prudence, whereby are confounded the machinations of all the villains and villainesses, wily cardinals, intriguing princes, "advocates" who forge signatures, ladies' maids who ransack drawers and steal documents. Everything comes right at last, and, through the assistance of a German Monte Christo, who endows him with a fortune, Lucio is able to marry the Princess's stepdaughter and live happy ever after.

